# Supplementary material for: Evaluation of phosphogypsum and pore volume water rates for reclaiming saline-sodic cambisols of Metehara sugar estate, Central Rift Valley of Ethiopia
Source: PLoS One. 2026 Sep 11;21(9):e0345688. doi: 10.1371/journal.pone.0345688 (PMC13567798; doi:10.1371/journal.pone.0345688)
Supplement: Supplementary file 1 [file pone.0345688.s001.docx]

S1 Table. Long-Term Mean Monthly Weather Data in Matahara Areas

| Month | Rainfall (mm) | Evapotranspiration (mm) | Temperature (^0^C) | | |
| --- | --- | --- | --- | --- | --- |
|  |  |  | Minimum | Maximum | Average |
| January | 15 | 130 | 15 | 31 | 23 |
| February | 33 | 127 | 16 | 32 | 24 |
| March | 50 | 128 | 18 | 34 | 26 |
| April | 42 | 123 | 19 | 34 | 27 |
| May | 36 | 124 | 19 | 35 | 27 |
| June | 28 | 113 | 21 | 36 | 29 |
| July | 122 | 109 | 20 | 33 | 27 |
| August | 132 | 115 | 20 | 32 | 26 |
| September | 46 | 119 | 19 | 33 | 26 |
| October | 26 | 126 | 16 | 33 | 25 |
| November | 8 | 128 | 14 | 31 | 23 |
| December | 7 | 130 | 13 | 30 | 22 |
| Annual | 553 | 1472 | 18 | 30 | 25 |

Source: Metehara meteorological data
