# Supplementary material for: Evaluation of phosphogypsum and pore volume water rates for reclaiming saline-sodic cambisols of Metehara sugar estate, Central Rift Valley of Ethiopia
Source: PLoS One. 2026 Sep 11;21(9):e0345688. doi: 10.1371/journal.pone.0345688 (PMC13567798; doi:10.1371/journal.pone.0345688)
Supplement: Supplementary file 2 [file pone.0345688.s002.docx]

S1 Text. Official Letter to Access Research Field

Written in local language (Amaharic language) and translated to English as follow

To Metehara Sugar Factory

Subject: Request for Cooperation

Research work on soil salinity detection and prevention is being planned and preparations are underway at Kesem Sugar Factory.

However, since the conditions in Kesem are not safe to conduct the experiment, we respectfully request you to provide the necessary support to the PhD students named below who are conducting this research, as it is planned to be implemented in fields with similar salinity problems at your factory (Metehara Sugar Factory).

1 Feredegn Dagnew

2 Gashaw Jifara

3 Kebenu Feyisa	I’m one of the individuals allowed to conduct the research on the field

4 Ashenafi Worku

5 Hagerenesh Sankura

With Regards

Director of Research and Development Center at Ethiopian Sugar Corporation
