# Supplementary material for: Evaluation of phosphogypsum and pore volume water rates for reclaiming saline-sodic cambisols of Metehara sugar estate, Central Rift Valley of Ethiopia
Source: PLoS One. 2026 Sep 11;21(9):e0345688. doi: 10.1371/journal.pone.0345688 (PMC13567798; doi:10.1371/journal.pone.0345688)
Supplement: Supplementary file 3 [file pone.0345688.s003.docx]

S2 Table. Raw Data of Licheate Analysis

| Column Experiment Raw Data For Analysis | | | | | | | | | |
| --- | --- | --- | --- | --- | --- | --- | --- | --- | --- |
| Treatment Rates | PV Rates | Replication | pH | Ece | Ca | Mg | Na | K | SAR |
| 0%GR | 1 | 1 | 8.4 | 11.1 | 1.8 | 0.13 | 23.3 | 0.1 | 23.7 |
| 0%GR | 1 | 2 | 8.3 | 9.8 | 1.9 | 0.12 | 22.9 | 0.1 | 22.8 |
| 0%GR | 1 | 3 | 8.1 | 11.0 | 1.8 | 0.12 | 23.0 | 0.2 | 23.5 |
|  |  |  |  |  |  |  |  |  |  |
| 0%GR | 2 | 1 | 8.7 | 7.0 | 1.8 | 0.13 | 23.1 | 0.2 | 23.5 |
| 0%GR | 2 | 2 | 8.4 | 8.0 | 1.8 | 0.13 | 23.3 | 0.1 | 23.7 |
| 0%GR | 2 | 3 | 8.5 | 7.6 | 1.9 | 0.13 | 23.2 | 0.1 | 23.0 |
|  |  |  |  |  |  |  |  |  |  |
| 0%GR | 3 | 1 | 8.8 | 7.6 | 1.5 | 0.11 | 23.4 | 0.1 | 25.2 |
| 0%GR | 3 | 2 | 8.7 | 7.7 | 1.8 | 0.14 | 23.3 | 0.1 | 24.5 |
| 0%GR | 3 | 3 | 8.6 | 7.6 | 1.7 | 0.13 | 23.7 | 0.2 | 24.8 |
|  |  |  |  |  |  |  |  |  |  |
| 0%GR | 4 | 1 | 8.7 | 7.4 | 1.5 | 0.11 | 23.4 | 0.2 | 26.1 |
| 0%GR | 4 | 2 | 8.7 | 7.5 | 1.5 | 0.13 | 23.7 | 0.1 | 26.3 |
| 0%GR | 4 | 3 | 8.8 | 7.9 | 1.5 | 0.12 | 23.6 | 0.2 | 26.2 |
|  |  |  |  |  |  |  |  |  |  |
| 0%GR | 5 | 1 | 8.4 | 7.7 | 1.4 | 0.12 | 23.8 | 0.1 | 27.3 |
| 0%GR | 5 | 2 | 8.3 | 7.3 | 1.6 | 0.13 | 24.0 | 0.1 | 25.8 |
| 0%GR | 5 | 3 | 8.5 | 7.4 | 1.5 | 0.12 | 24.0 | 0.1 | 26.7 |
|  |  |  |  |  |  |  |  |  |  |
| 50%GR | 1 | 1 | 8.4 | 10.6 | 9.4 | 0.7 | 21.2 | 0.2 | 9.5 |
| 50%GR | 1 | 2 | 8.2 | 11.0 | 9.0 | 0.5 | 20.7 | 0.1 | 8.2 |
| 50%GR | 1 | 3 | 8.4 | 10.2 | 9.3 | 0.8 | 20.8 | 0.1 | 10.1 |
|  |  |  |  |  |  |  |  |  |  |
| 50%GR | 2 | 1 | 8.4 | 6.3 | 10.9 | 0.9 | 19.4 | 0.1 | 8.5 |
| 50%GR | 2 | 2 | 8.1 | 6.0 | 10.3 | 1.2 | 19.0 | 0.2 | 8.0 |
| 50%GR | 2 | 3 | 8.2 | 5.8 | 10.6 | 1.0 | 19.1 | 0.1 | 7.5 |
|  |  |  |  |  |  |  |  |  |  |
| 50%GR | 3 | 1 | 8.2 | 5.9 | 10.2 | 0.8 | 17.5 | 0.2 | 7.4 |
| 50%GR | 3 | 2 | 7.9 | 5.4 | 10.0 | 1.1 | 16.6 | 0.1 | 7.1 |
| 50%GR | 3 | 3 | 8.2 | 5.7 | 9.8 | 1.2 | 17.0 | 0.1 | 7.1 |
|  |  |  |  |  |  |  |  |  |  |
| 50%GR | 4 | 1 | 8.0 | 6.2 | 11.4 | 1.0 | 15.7 | 0.1 | 6.5 |
| 50%GR | 4 | 2 | 8.1 | 5.9 | 11.3 | 1.2 | 15.8 | 0.2 | 6.3 |
| 50%GR | 4 | 3 | 8.4 | 6.0 | 11.3 | 1.1 | 15.6 | 0.2 | 6.2 |
|  |  |  |  |  |  |  |  |  |  |
| 50%GR | 5 | 1 | 7.9 | 5.5 | 12.6 | 0.7 | 14.4 | 0.1 | 5.8 |
| 50%GR | 5 | 2 | 8.2 | 5.8 | 12.6 | 0.9 | 14.5 | 0.3 | 5.5 |
| 50%GR | 5 | 3 | 8.0 | 5.7 | 12.4 | 1.0 | 14.6 | 0.1 | 5.4 |
|  |  |  |  |  |  |  |  |  |  |
| 75%GR | 1 | 1 | 8.5 | 10.8 | 9.3 | 0.7 | 24.8 | 0.3 | 12.5 |
| 75%GR | 1 | 2 | 8.3 | 10.4 | 9.5 | 1.0 | 25.7 | 0.6 | 10.1 |
| 75%GR | 1 | 3 | 8.4 | 10.6 | 9.5 | 0.9 | 26.0 | 0.5 | 9.6 |
|  |  |  |  |  |  |  |  |  |  |
| 75%GR | 2 | 1 | 8.3 | 8.6 | 10.0 | 1.4 | 20.0 | 0.2 | 8.5 |
| 75%GR | 2 | 2 | 8.3 | 8.3 | 9.7 | 2.0 | 19.8 | 0.4 | 9.3 |
| 75%GR | 2 | 3 | 8.3 | 8.5 | 9.9 | 2.1 | 20.4 | 0.3 | 7.2 |
|  |  |  |  |  |  |  |  |  |  |
| 75%GR | 3 | 1 | 8.1 | 6.3 | 10.5 | 1.6 | 15.9 | 0.1 | 6.9 |
| 75%GR | 3 | 2 | 8.4 | 6.0 | 10.7 | 1.5 | 16.2 | 0.2 | 6.6 |
| 75%GR | 3 | 3 | 8.2 | 5.8 | 10.9 | 1.4 | 16.0 | 0.3 | 7.0 |
|  |  |  |  |  |  |  |  |  |  |
| 75%GR | 4 | 1 | 8.0 | 6.4 | 12.0 | 0.9 | 13.2 | 0.1 | 5.2 |
| 75%GR | 4 | 2 | 8.2 | 7.3 | 12.3 | 1.0 | 13.5 | 0.3 | 5.1 |
| 75%GR | 4 | 3 | 8.1 | 6.6 | 12.1 | 1.1 | 13.2 | 0.2 | 5.3 |
|  |  |  |  |  |  |  |  |  |  |
| 75%GR | 5 | 1 | 7.8 | 5.2 | 13.0 | 0.9 | 11.5 | 0.1 | 4.8 |
| 75%GR | 5 | 2 | 8.0 | 5.0 | 12.6 | 1.0 | 11.0 | 0.2 | 4.2 |
| 75%GR | 5 | 3 | 8.2 | 5.1 | 12.6 | 0.7 | 11.1 | 0.1 | 4.0 |
|  |  |  |  |  |  |  |  |  |  |
| 100%GR | 1 | 1 | 8.0 | 10.1 | 10.7 | 0.6 | 26.7 | 0.1 | 12.2 |
| 100%GR | 1 | 2 | 8.3 | 10.3 | 10.2 | 1.0 | 26.4 | 0.1 | 10.1 |
| 100%GR | 1 | 3 | 8.3 | 10.0 | 10.6 | 1.2 | 26.4 | 0.4 | 11.1 |
|  |  |  |  |  |  |  |  |  |  |
| 100%GR | 2 | 1 | 8.0 | 5.8 | 12.3 | 1.5 | 20.5 | 0.2 | 7.3 |
| 100%GR | 2 | 2 | 8.2 | 6.1 | 12.0 | 1.2 | 20.8 | 0.7 | 7.5 |
| 100%GR | 2 | 3 | 8.4 | 5.9 | 12.0 | 1.3 | 20.2 | 0.6 | 8.9 |
|  |  |  |  |  |  |  |  |  |  |
| 100%GR | 3 | 1 | 7.7 | 4.6 | 13.7 | 1.5 | 16.4 | 0.1 | 7.4 |
| 100%GR | 3 | 2 | 7.9 | 4.6 | 13.6 | 1.3 | 17.2 | 0.1 | 5.0 |
| 100%GR | 3 | 3 | 8.2 | 4.5 | 13.2 | 1.1 | 16.9 | 0.4 | 6.2 |
|  |  |  |  |  |  |  |  |  |  |
| 100%GR | 4 | 1 | 7.8 | 3.6 | 13.2 | 0.7 | 13.5 | 0.1 | 5.9 |
| 100%GR | 4 | 2 | 7.6 | 3.0 | 12.6 | 0.9 | 13.2 | 0.1 | 4.4 |
| 100%GR | 4 | 3 | 8.0 | 3.0 | 12.9 | 1.1 | 13.5 | 0.1 | 5.0 |
|  |  |  |  |  |  |  |  |  |  |
| 100%GR | 5 | 1 | 7.6 | 3.2 | 14.3 | 0.8 | 12.4 | 0.1 | 4.1 |
| 100%GR | 5 | 2 | 7.9 | 3.3 | 14.6 | 1.2 | 12.8 | 0.4 | 4.3 |
| 100%GR | 5 | 3 | 7.6 | 2.8 | 14.0 | 1.0 | 12.3 | 0.2 | 4.0 |
|  |  |  |  |  |  |  |  |  |  |
| 150%GR | 1 | 1 | 8.0 | 10.0 | 11.0 | 1.0 | 27.5 | 0.1 | 11.4 |
| 150%GR | 1 | 2 | 8.4 | 9.5 | 11.6 | 1.2 | 27.8 | 0.3 | 12.5 |
| 150%GR | 1 | 3 | 8.2 | 9.6 | 11.3 | 1.1 | 28.3 | 0.2 | 11.8 |
|  |  |  |  |  |  |  |  |  |  |
| 150%GR | 2 | 1 | 8.2 | 4.7 | 12.7 | 1.0 | 18.5 | 0.1 | 6.3 |
| 150%GR | 2 | 2 | 8.5 | 4.2 | 11.4 | 1.4 | 17.8 | 0.4 | 7.1 |
| 150%GR | 2 | 3 | 8.2 | 4.9 | 12.2 | 1.2 | 18.3 | 0.1 | 8.0 |
|  |  |  |  |  |  |  |  |  |  |
| 150%GR | 3 | 1 | 8.2 | 4.2 | 14.3 | 1.6 | 14.3 | 0.2 | 4.9 |
| 150%GR | 3 | 2 | 7.5 | 4.7 | 13.5 | 2.0 | 13.3 | 0.6 | 4.8 |
| 150%GR | 3 | 3 | 8.0 | 4.6 | 13.6 | 1.8 | 13.2 | 0.7 | 4.9 |
|  |  |  |  |  |  |  |  |  |  |
| 150%GR | 4 | 1 | 8.0 | 2.9 | 14.8 | 1.1 | 9.8 | 0.1 | 3.3 |
| 150%GR | 4 | 2 | 7.7 | 2.7 | 13.8 | 1.5 | 10.5 | 0.3 | 3.5 |
| 150%GR | 4 | 3 | 7.7 | 2.1 | 13.7 | 1.6 | 10.0 | 0.2 | 4.0 |
|  |  |  |  |  |  |  |  |  |  |
| 150%GR | 5 | 1 | 8.0 | 1.9 | 14.6 | 1.5 | 7.2 | 0.1 | 0.9 |
| 150%GR | 5 | 2 | 7.5 | 2.1 | 15.2 | 0.8 | 7.8 | 0.2 | 1.0 |
| 150%GR | 5 | 3 | 7.6 | 2.3 | 14.0 | 1.3 | 7.2 | 0.1 | 1.1 |
|  |  |  |  |  |  |  |  |  |  |
| 200%GR | 1 | 1 | 8.0 | 11.3 | 11.5 | 0.6 | 33.0 | 0.1 | 12.8 |
| 200%GR | 1 | 2 | 8.4 | 10.4 | 12.4 | 1.0 | 33.8 | 0.3 | 14.4 |
| 200%GR | 1 | 3 | 8.5 | 10.1 | 11.5 | 1.1 | 33.1 | 0.2 | 12.4 |
|  |  |  |  |  |  |  |  |  |  |
| 200%GR | 2 | 1 | 8.4 | 4.0 | 12.4 | 0.6 | 16.8 | 0.1 | 6.8 |
| 200%GR | 2 | 2 | 8.0 | 4.4 | 12.9 | 1.0 | 16.0 | 0.3 | 5.5 |
| 200%GR | 2 | 3 | 8.2 | 4.2 | 13.0 | 1.1 | 15.8 | 0.2 | 7.0 |
|  |  |  |  |  |  |  |  |  |  |
| 200%GR | 3 | 1 | 8.0 | 3.9 | 13.7 | 1.0 | 12.2 | 0.3 | 4.5 |
| 200%GR | 3 | 2 | 7.7 | 3.6 | 14.2 | 1.3 | 12.7 | 0.1 | 5.1 |
| 200%GR | 3 | 3 | 8.0 | 3.9 | 13.8 | 1.0 | 12.9 | 0.2 | 4.2 |
|  |  |  |  |  |  |  |  |  |  |
| 200%GR | 4 | 1 | 7.7 | 2.8 | 14.5 | 1.0 | 6.0 | 0.2 | 2.5 |
| 200%GR | 4 | 2 | 7.9 | 2.0 | 13.7 | 1.4 | 6.2 | 0.7 | 2.6 |
| 200%GR | 4 | 3 | 7.5 | 2.4 | 13.8 | 1.2 | 6.8 | 0.3 | 2.1 |
|  |  |  |  |  |  |  |  |  |  |
| 200%GR | 5 | 1 | 7.3 | 1.8 | 14.5 | 1.4 | 4.8 | 0.2 | 0.8 |
| 200%GR | 5 | 2 | 7.7 | 2.1 | 15.0 | 1.0 | 5.2 | 0.5 | 0.9 |
| 200%GR | 5 | 3 | 7.5 | 2.1 | 14.3 | 1.5 | 5.3 | 0.2 | 1.0 |
|  |  |  |  |  |  |  |  |  |  |
| 100%gypsum | 1 | 1 | 8.0 | 10.2 | 10.8 | 0.9 | 25.0 | 0.2 | 9.0 |
| 100%gypsum | 1 | 2 | 8.3 | 10.0 | 11.3 | 1.0 | 25.4 | 0.5 | 11.2 |
| 100%gypsum | 1 | 3 | 8.3 | 10.2 | 10.9 | 1.1 | 24.9 | 0.2 | 10.4 |
|  | |  |  |  |  |  |  |  |  |
| 100%gypsum | 2 | 1 | 8.2 | 6.8 | 12.3 | 1.0 | 21.2 | 0.2 | 8.3 |
| 100%gypsum | 2 | 2 | 8.0 | 6.5 | 11.6 | 1.4 | 20.7 | 0.1 | 7.9 |
| 100%gypsum | 2 | 3 | 8.4 | 6.5 | 11.8 | 1.2 | 20.8 | 0.3 | 8.4 |
|  | |  |  |  |  |  |  |  |  |
| 100%gypsum | 3 | 1 | 7.6 | 4.8 | 13.8 | 1.3 | 17.9 | 0.4 | 6.0 |
| 100%gypsum | 3 | 2 | 8.0 | 5.3 | 13.0 | 1.1 | 17.6 | 0.4 | 6.7 |
| 100%gypsum | 3 | 3 | 8.1 | 4.9 | 13.1 | 1.5 | 17.6 | 0.1 | 6.8 |
|  | |  |  |  |  |  |  |  |  |
| 100%gypsum | 4 | 1 | 7.9 | 4.8 | 14.5 | 1.2 | 14.9 | 0.3 | 5.2 |
| 100%gypsum | 4 | 2 | 7.7 | 4.5 | 13.8 | 0.7 | 14.4 | 0.7 | 5.4 |
| 100%gypsum | 4 | 3 | 7.5 | 4.5 | 13.7 | 0.8 | 14.1 | 0.5 | 5.3 |
| 100%gypsum | 5 | 1 | 7.7 | 3.9 | 14.8 | 0.9 | 7.9 | 0.3 | 5.2 |
| 100%gypsum | 5 | 2 | 7.4 | 3.5 | 14.0 | 0.8 | 7.6 | 0.2 | 4.5 |
| 100%gypsum | 5 | 3 | 7.4 | 3.4 | 14.1 | 1.3 | 7.3 | 0.1 | 4.8 |

*Where PV is pore volume of leaching water, ECe is electrical conductivity at saturated extract, SAR is sodium adsorption ration of the licheate solution.*
