# Supplementary material for: Evaluation of phosphogypsum and pore volume water rates for reclaiming saline-sodic cambisols of Metehara sugar estate, Central Rift Valley of Ethiopia
Source: PLoS One. 2026 Sep 11;21(9):e0345688. doi: 10.1371/journal.pone.0345688 (PMC13567798; doi:10.1371/journal.pone.0345688)
Supplement: Supplementary file 4 [file pone.0345688.s005.docx]

**Author Responses to the Comments Provided By Academic Editor and Reviewers**

**A. Responses to the comments provided by Academic editor**

1. Yes, the manuscript developed based on PLOS ONE's style requirements, including those for file naming.

2. Access to the field site was approved by “**Director of Research and Development Center at Ethiopian Sugar Corporation**” the official letter is attached under supporting information file”

3. "The funders had no role in study design, data collection and analysis, decision to publish, or preparation of the manuscript."

4. Except in the Funding Statement section, any funding-related text was removed from other part of the manuscript.

5. "This does not alter our adherence to PLOS ONE policies on sharing data and materials.”

6. The unpublished and not yet been accepted for publication data sources were removed

7 Figure 1 changed and replaced with Figure extracted from “USGS National Map Viewer (public domain): <http://viewer.nationalmap.gov/viewer/>” as per the given recommendation

8. Captions for Supporting Information files were provided at the end of the manuscript.

9. specific Supplementary table included in the 'Supporting Information.

10. Reviewer #1 commented to access research reports done in China on PG and we used some already
11. We reviewed the reference list to ensure that it is complete and correct.

**B. Responses to the comments provided by Reviewer #1**

(1) **Yes**, we have used already some of the research findings done in china

(2) Ok, abbreviations were checked

(3) Yes, units/dimensions corresponding to the formulas and symbols were checked and revised.

(4) It is to mean 88% Gypsum purity. Gypsum is a naturally occurring soft mineral composed primarily of calcium sulfate dihydrate (CaSO₄·2H₂O). This purity rating means that 88% of the material consists of effective gypsum, while the remaining 12% is made up of impurities such as clay, silica, limestone, and other naturally occurring minerals.

(5) It is true Column leaching tests only examined the effect of phosphogypsum on improving soil salinity and other indicators in the controlled environment. To know its applicability, the field test is recommended. From our side we have an extension work of this in the field which is ongoing currently.

(6) The presence of harmful impurities in phosphogypsum necessitates careful consideration of its potential environmental impacts. Outbakat et al. (2022) reported no negative effects associated with the use of phosphogypsum for the reclamation of salt-affected soils in Moroccan conditions. In the present study, phosphogypsum obtained from the same source was used. However, the environmental implications of its application under Ethiopian soil conditions require further investigation. Studies addressing this issue are currently being undertaken by our colleagues to evaluate its suitability and potential risks in Ethiopian agro-ecosystems.

**C. Responses to the comments provided by Reviewer #2**
1. It is accepted that the treatment combination were seven levels of PG application and five levels of leaching water (PV) , which result in 35 treatment combinations.

2. It is true that many researches were done in the other parts of the globe, but in Ethiopia it is very rare and so that PG’s different level in combination with different leaching water volumes were analyzed targeting on region-specific area (Metehara sugar state farm site).

3. In agricultural and soil science experiments, a commonly accepted guideline is that the error degrees of freedom (dfe) should be at least 12, and preferably above 20, to provide sufficient statistical power and a dependable estimate of experimental error. In the present study, which employed a Completely Randomized Design (CRD), the error degrees of freedom were calculated as:

dfe=Total treatment combinations×(r−1)

With 35 treatment combinations and three replications (r=3):

dfe=35×(3−1)=70

Thus, the experiment generated 70 error degrees of freedom, which is substantially higher than the recommended minimum range of 12–20. This large number of error degrees of freedom enhances the precision of error variance estimation and provides very high statistical power for detecting even small treatment effects and interactions between phosphogypsum (PG) application rates and leaching water levels.

4. The unpublished data sources were removed and replaced with formal once and also the data acquisition process was described clearly.
5. In the manuscript it had been tried discussion of the practical significance of the results

6. Figures 4 and 5 were revised and error bars were added for model fitting to show the **variability, uncertainty, or margin of error** in the data

7. we tried to apply Professional English editing is recommended.
8. The Introduction revised and condensed by targeting to research questions/objectives

9. The manuscript tried to provide supporting data on the properties of PG within the study.

10. Actually, this research conducted in controlled environment (in laboratory). It is recommended to be repeated in green or lath houses and then based on those findings be tested in the field.
